# Drowning of a patient with epilepsy while showering

**DOI:** 10.1186/s12199-019-0792-x

**Published:** 2019-05-13

**Authors:** Risako Nakagawa, Wataru Ishii, Masahito Hitosugi

**Affiliations:** Department of Legal Medicine, Shiga University of Medical Science, Seta-Tsukinowa-cho, Otsu, Shiga 520-2192 Japan

**Keywords:** Epilepsy, Nonadherence, Drowning, Shower, Autopsy, Prevention

## Abstract

In Japan, because the most common site of drowning among patients with epilepsy is the bathtub, showering is generally recommended as an alternative to bathing. We herein report a case involving a female patient with epilepsy who drowned while showering. She had been diagnosed with epilepsy approximately 25 years previously, and her condition had progressed to refractory epilepsy. Carbamazepine, levetiracetam, lamotrigine, clobazam, and perampanel were prescribed daily. One day while showering, the patient was found lying with her face immersed in water that had accumulated on the floor of the bathtub. A forensic autopsy revealed water in the stomach, trachea, and proximal regions of both lung bronchi as well as white and red foam on the pharynx and larynx. A total of 1.9 μg/mL of lamotrigine, 0.14 μg/mL of carbamazepine, and 0.069 μg/mL of perampanel were detected in the patient’s blood. The patient’s cause of death was determined to be drowning due to an epileptic seizure. Although the patient was prescribed five types of antiepileptic medication, only three were detected in her blood. The current case demonstrates that drowning can occur while showering, suggesting that it is unsafe for patients with medication nonadherence. To prevent unintentional deaths in the bathroom, we recommend that patients with epilepsy maintain high adherence to all prescriptions and are supervised by a family member, even when showering. The current case is the first autopsy report of a patient with epilepsy who drowned while showering.

Dear Editor,

A previous report in this journal concerning the seasonality of injury-related mortality in children and adolescents in Japan revealed that the incidence of drowning deaths increased especially in summer because many children and adolescents play in rivers or the sea during summer vacation [1]. However, because approximately 40% of accidental drowning deaths among children occur in bathtubs [2], prevention of drowning while bathing should be promoted.

Patients with epilepsy have traditionally been considered to have a higher risk of death due to drowning. A meta-analysis based on 51 cohorts suggested that the risk of drowning among patients with epilepsy is 15- to 19-fold higher than that in the general population [3]. In Japan, bathing habits traditionally involve immersion in hot water almost daily. A previous study suggested that the relative risk of drowning in the bathtub is 96 times higher in children with than without epilepsy [3]. To ameliorate this risk, patients with epilepsy should use showers rather than immersion in water for bathing. The Epilepsy Information Center in Japan recommends that patients with epilepsy should have showers rather than baths to reduce the risk of drowning [4]. As a result, many Japanese patients with epilepsy, particularly young people and their family members, believe that showers are safer than baths. However, we herein describe a patient with epilepsy who drowned while showering. Thus, although patients are not immersed in water while showering, drowning is still possible, especially in patients with nonadherence to medications. This case highlights the importance of maintaining adherence to prevent unintentional drowning deaths even when not immersed in water.

A woman in her 30s had been diagnosed with epilepsy approximately 25 years previously, and her condition progressed to refractory epilepsy. The following medications were prescribed: 200 mg of carbamazepine, 250 mg of levetiracetam, and 100 mg of lamotrigine twice daily (every morning and evening); 10 mg of clobazam once every morning; and 2 mg of perampanel once before bed. However, the patient developed epileptic seizures during the month before her death, with the last seizure attack occurring 4 days before her death. The patient lived with her two children, a boy and girl of elementary school age. On the day of her death, after the two children had left the house to go to school, the patient went to the bathroom and showered around 10:00 AM. When her mother visited the house around 2:30 PM, the patient was found lying on the floor, outside of the bathtub. The room temperature was 16 °C. Because the water was flowing from the showerhead and the patient was in the supine position with her body covering the drain outlet, her face was immersed in water that had accumulated on the floor. Although paramedics arrived soon thereafter, the patient had already developed rigor mortis and was declared dead. A forensic autopsy was performed the next day to clarify the cause of death. No evidence of trauma was present on the surface of the skin. The autopsy revealed findings of acute death: fluidity of blood, visceral congestion, and conjunctival petechiae. White and red foam were found on the pharynx and larynx, and moderate amounts of water were found in the trachea and proximal regions of both lung bronchi. The heart weighed 387.2 g and exhibited no abnormal findings. The autopsy revealed 420 ml of water in the stomach. The brain weighed 1436 g and exhibited edema and congestion but was otherwise normal. Toxicologically, no alcohol was detected in the blood or urine. The following drugs were detected in the blood using liquid chromatography-mass spectrometry: 1.9 μg/mL of lamotrigine, 0.14 μg/mL of carbamazepine, 0.069 μg/mL of perampanel, and 0.026 μg/mL of diazepam. The cause of death was determined to be drowning due to an epileptic seizure.

Before this event, the patient had been prescribed five types of antiepileptic medicine for the treatment of refractory epilepsy. However, only three of the prescribed antiepileptic drugs were detected in the patient’s blood. Moreover, the blood concentration of perampanel was below the therapeutic range. The blood concentrations of the drugs were not considered to have decreased after death because these drugs are not decomposed by blood enzymes after death. This indicates that the patient had not taken the epileptic medications as prescribed. This nonadherence was in accordance with the finding that the patient had developed frequent seizure attacks within the month before her death. The overall nonadherence rate of patients prescribed antiepileptic drugs reportedly ranges from 30 to 50%, contributing to an increased risk of epileptic seizures [5]. Therefore, it is important that patients with epilepsy maintain a high adherence rate. Because the most common site of drowning among patients with epilepsy is the bathtub, showering instead of bathing is generally recommended [6]. However, because showering can lead to drowning as described in the present case, it is also unsafe for patients with nonadherence. To prevent unintentional deaths in the bathroom, we recommend that a family member supervises patients with epilepsy when showering. Alternatively, patients with epilepsy should shower only when a family member is residing in the house.

The worldwide lifetime prevalence of epilepsy is approximately 0.8% [7], and the estimated number of patients with epilepsy in Japan ranges from 650,000 to 1 million [8]. We have herein reported the first autopsy case of a patient with epilepsy who drowned while showering, highlighting the importance of maintaining adherence and supervision while bathing.
